# Correction to “Enhanced Biomechanical Stability in Proximal Humeral Fractures: Finite Element Analysis of a Novel Endosteal Anatomical Support Nail for Improved Fixation in Elderly Patients”

**DOI:** 10.1111/os.70106

**Published:** 2025-06-18

J. Chen, Z. Wang, C. Li, et al., “Enhanced Biomechanical Stability in Proximal Humeral Fractures: Finite Element Analysis of a Novel Endosteal Anatomical Support Nail for Improved Fixation in Elderly Patients,” *Orthopaedic Surgery* 17, no. 2 (2025): 551–562, https://doi.org/10.1111/os.14297.

The affiliation listing for Changda Li: “2 (The Department of Orthopaedic Surgery, Fourth Medical Center for Chinese PLA General Hospital, Beijing, China), 3 (Ningxia Medical University, Yinchuan, China)” was listed incorrectly.

The correct affiliation should be: “Changda Li: 3 (Ningxia Medical University, Yinchuan, China).”

We apologize for this error and any inconvenience it may have caused.

